# Multiple Evanescent White Dot Syndrome (MEWDS) Following COVID-19 Infection: A Presumed Recurrence

**DOI:** 10.7759/cureus.104405

**Published:** 2026-02-27

**Authors:** Mohammed Mehdi Shahid, Nikki N Neequaye, Amde Selassie Shifera

**Affiliations:** 1 Ophthalmology, University of Rochester School of Medicine and Dentistry, Rochester, USA; 2 Ophthalmology, University of Rochester Medical Center, Rochester, USA

**Keywords:** case report, covid-19, multiple evanescent white dot syndrome, ophthalmology, retina, white dot syndrome

## Abstract

Multiple evanescent white dot syndrome (MEWDS) is a transient self-limiting likely post-viral inflammatory condition involving the outer retina and inner choroid and has been reported following several vaccinations and viral infections, including COVID-19. Photopsias are a common presenting symptom but rarely persist after the acute phase of MEWDS. We present a case of an otherwise healthy 29-year-old woman who developed persistent photopsias of the right eye following COVID-19 infection. These photopsias persisted for several months and worsened after a second COVID-19 infection, which is atypical of MEWDS. Fundus autofluorescence (FAF) demonstrated several characteristic punctate hyperautofluorescent spots. Her symptoms and fundus lesions resolved after a few weeks of diagnosis without treatment. COVID-19 has several reported ocular manifestations, including MEWDS. While most cases of MEWDS following COVID-19 infection are singular instances, our case was a presumed recurrence. MEWDS is often self-limiting, but in this case, symptoms persisted for several months, suggesting the possibility of long COVID, which is a poorly understood phenomenon.

## Introduction

Multiple evanescent white dot syndrome (MEWDS) is a relatively rare, transient, and self-limiting inflammatory reaction characterized by small, yellow-white lesions at the level of the outer retina or retinal pigment epithelium [1]. The typical presentation of MEWDS involves sudden onset unilateral diminution of vision, scotomas, and photopsias in myopic healthy women aged 20 to 50 years, even though it has also been reported in males and older subjects​ [1,2]. Although the pathogenesis of MEWDS is not fully understood, it has been associated in at least one-third of the cases with a viral prodrome that is suspected to trigger an autoimmune response. Several reports have described MEWDS following various vaccinations, including hepatitis A, hepatitis B, human papillomavirus (HPV), influenza, measles-mumps-rubella (MMR), varicella, rabies, and yellow fever, with a median onset of two weeks after vaccination [3-5]. Recently, there have been reports of MEWDS following both COVID-19 vaccination and infection​ [4,6-8].

MEWDS can manifest anywhere between a few weeks and several months following the immunologic trigger. The exact latency period is highly variable in post-infectious or post-vaccination cases that have been reported [3-8]. Multimodal imaging reveals characteristic hypopigmented lesions on fundus imaging. In fact, fundus fluorescein angiography and optical coherence tomography remain the mainstay diagnostic tools in the diagnosis of MEWDS [9]. A thorough systemic and infectious workup is essential to exclude other chorioretinopathies. Most cases resolve spontaneously or with corticosteroid therapy, and visual prognosis is favorable in most cases [1,8]. Recurrence of MEWDS is sporadic but distinctly rare, and has been documented, particularly in the setting of repeated immune stimulation such as subsequent COVID-19 vaccination or viral reactivation ​[5,6]. Herein, we present a case of MEWDS, presumed to be a recurrent episode, with the onset of symptoms two weeks after a second COVID-19 infection.

## Case presentation

The patient is a healthy Caucasian woman in her late 20s with mild myopia (-0.75 spherical diopters in both eyes). She first presented to her optometrist with the complaint of blurry vision and photopsias of the right eye (OD). The onset of these symptoms was two weeks after serologically confirmed COVID-19 infection. Examination was notable for vitreous syneresis of both eyes but was otherwise unremarkable, including a normal fundus exam. At that time, no multi-modal imaging was obtained, which retrospectively limited the diagnostic certainty of the first episode of MEWDS. The patient continued to have photopsias for the next eight months and subsequently developed worsening photopsias following a second COVID-19 infection. The patient then presented to the uveitis service two months later. At this point, her photopsias had persisted for 10 months after the initial onset, with worsening of symptoms after her second COVID-19 infection. On examination, best corrected visual acuity (BCVA) was 20/20 OD and 20/20 in her left eye (OS), and her intraocular pressures (IOP) were 20 mmHg in each eye. Slit lamp exam was unremarkable with a quiet anterior chamber and vitreous in both eyes.

**Figure 1:**
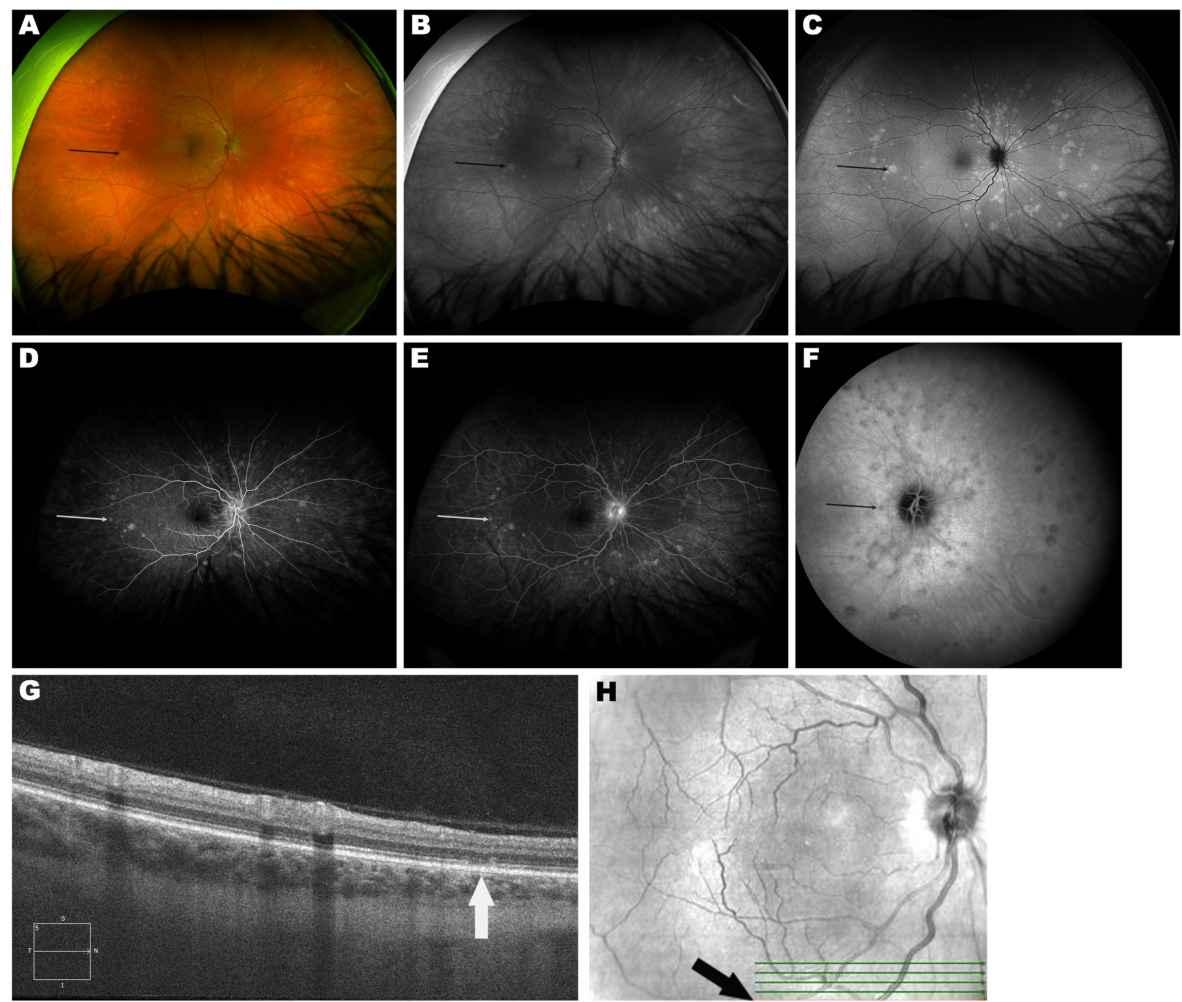
Multi-modal imaging of the right eye (OD) at first presentation. A: Ultra-wide-field pseudocolor fundus photograph showing small yellow spots (black arrow) in the temporal and nasal mid periphery and inferior and superior to the optic disc (Optos, Marlborough, MA, USA). B. Ultra-wide field fundus photograph, green channel (Optos) showing the fundus lesions as white spots (black arrow). C: Fundus autofluorescence showing numerous punctate hyperautofluorescent spots (black arrow). D and E: Fluorescein angiography showing numerous spots of early hyperfluorescence (white arrows). F: Indocyanine green angiography showing numerous hypofluorescent patches (black arrow). G and H: Spectral Domain Optical Coherence Tomography (Heidelberg, Franklin, MA, USA) showing focal disruption of ellipsoid zone (G) at one of the lesion sites (white arrow) with location of the scan shown in H (black arrow).

Retinal examination of the right eye showed several small yellow spots in the temporal and nasal midperiphery and inferior and superior to the optic disc (Figures 1A, 1B). Fundus autofluorescence (FAF) of the right eye revealed characteristic multiple punctate hyperautofluorescent spots in the temporal and nasal midperiphery and inferior and superior to the optic disc (Figure 1C). Fluorescein angiography (FA) of the right eye showed numerous spots of early hyperfluorescence (Figures 1D, 1E), while on indocyanine green angiography (ICGA), there were several hypofluorescent spots (Figure 1F). Spectral domain optical coherence tomography (OCT) showed focal ellipsoid zone disruption at the site of one of the lesions (Figures 1G, 1H). Laboratory testing, including complete blood cell count, antinuclear antibody (ANA), antineutrophil cytoplasmic antibody (ANCA), C-reactive protein (CRP), rheumatoid factor (RF), QuantiFERON-TB Gold (Qiagen, Germantown, USA), rapid plasma reagin (RPR), and treponema pallidum particle agglutination (TPPA), was carried out. These tests were found to be normal, excluding an elevated RF of 64 IU/mL (normal range: <14 IU/mL). The elevated RF was non-contributory to the case and purely incidental. Further rheumatologic evaluation was not pursued.

**Figure 2:**
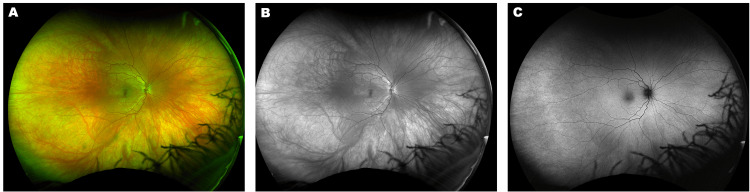
Resolution of MEWDS fundus lesions at four weeks follow-up. A and B: Fundus photograph, pseudocolor (A) and green channel (B) (Optos, Marlborough, MA, USA). C: Fundus autofluorescence (Optos). MEWDS: multiple evanescent white dot syndrome

A diagnosis of MEWDS of the right eye was made and was presumed to be triggered by her second COVID-19 infection. Alternative diagnoses of acute posterior multifocal placoid pigment epitheliopathy (APMPPE), birdshot choroidopathy, and multifocal choroiditis were considered but were ultimately excluded given the acute unilateral presentation and characteristic hyperautofluorescent spots on FAF, which are more typical of MEWDS [1]. AMPPE is distinguished by larger, bilateral placoid lesions at the level of the retinal pigment epithelium, while birdshot choroidopathy presents with bilateral cream-colored choroidal lesions in an insidious course [1]. Multifocal choroiditis presents bilaterally with characteristic punched-out chorioretinal scars [1]. No treatment was given at the time of diagnosis. On follow-up examination four weeks later, her photopsias had reduced in frequency, now occurring only occasionally. Her fundus exam at follow-up was normal (Figures 2A, 2B), and imaging showed resolution of the hyperautofluorescent spots on FAF (Figure 2C).

## Discussion

This case is notable for two key features: persistent photopsias and presumed recurrence of MEWDS following a second COVID-19 infection. Photopsias are a common presenting symptom in MEWDS; however, their persistence beyond the acute phase is rare and has only been sporadically reported in the literature. Most patients experience resolution of positive visual phenomena as the retinal lesions resolve. Persistent photopsias are more commonly associated with atypical or severe cases. The spectrum of MEWDS has expanded and now includes visual snow and other positive phenomena, but these remain infrequent and poorly characterized​ [10,11]. Importantly, given the self-limiting and acute nature of MEWDS, recurrent cases are only sporadically mentioned in the literature. The lack of objective imaging during the initial presentation, therefore, suggests an alternative explanation that the patient’s course may have represented a single prolonged or biphasic inflammatory episode with fluctuating symptoms rather than true recurrence.

There are several case reports of ocular complications of COVID-19, including anterior uveitis, multifocal choroiditis, and panuveitis​ [12]. MEWDS has been reported in association with COVID-19 vaccination and rarely with COVID-19 infection​ [4,8,13-16]. Most reported cases of MEWDS have been singular instances, with recurrence being a rare phenomenon. Soifer et al. reported a case of recurrent MEWDS following the first dose and a booster of COVID-19 vaccination ​[6]. In addition, Ng & Niederer reported a case of recurrent MEWDS with the first episode occurring after COVID-19 vaccination and the second episode following a COVID-19 infection​ [7]. These cases of recurrent MEWDS, along with this case, implicate both direct viral effects and post-infectious immune dysregulation as possible mechanisms. The temporal association between the second COVID-19 infection and MEWDS recurrence in this case supports a hypothesis of immune-mediated pathogenesis, consistent with observations in vaccine-associated cases ​[3,4,8,9,17]. 

While the first instance of MEWDS in our case was presumed and not formally confirmed with multimodal imaging, the clinical history is suggestive of MEWDS with prolonged photopsias following the initial COVID-19 infection. We hypothesize that this association is suggestive of long COVID, which is a poorly understood condition and is recognized to affect multiple organ systems, including the retina​ [18,19]. This hypothesis is purely speculative as causality cannot be inferred from a single case. Long COVID can present with persistent neurological and neuro-ophthalmological symptoms, including visual disturbances, even in the absence of severe acute illness or systemic findings​ [20]. The mechanism is hypothesized to be through persistent release of cytokines, causing chronic low-grade inflammation ​[21]. In this case, no systemic inflammatory markers or cytokine profiling were available, which limits mechanistic inference. The relapsing-remitting nature of long COVID, especially following reinfection, may have contributed to symptom persistence and worsening in this case. However, causal inference between COVID-19 infection and MEWDS recurrence remains constrained in this case due to the lack of multimodal imaging during the first episode, retrospective inference of recurrence, and absence of objective biomarkers.

## Conclusions

In conclusion, this case adds to the growing body of evidence that MEWDS may present with persistent photopsias and recur in the setting of repeated immune stimulation, such as sequential COVID-19 infections. It underscores the need for heightened clinical vigilance and long-term follow-up in patients with atypical visual symptoms or a history of MEWDS, particularly in the context of ongoing viral exposure or immunization. The clinical course also highlights the importance of multimodal imaging and exclusion of alternative etiologies. The combination of persistent photopsias and presumed recurrence after a second COVID-19 infection represents a rare and clinically significant variant of MEWDS, with implications for diagnosis, management, and understanding of disease pathophysiology in the post-pandemic era.

## References

[1] Crawford CM, Igboeli O (2013). A review of the inflammatory chorioretinopathies: the white dot syndromes. ISRN Inflamm.

[2] Abu-Yaghi NE, Hartono SP, Hodge DO, Pulido JS, Bakri SJ (2011). White dot syndromes: a 20-year study of incidence, clinical features, and outcomes. Ocul Immunol Inflamm.

[3] Stangos A, Zaninetti M, Petropoulos I, Baglivo E, Pournaras C (2006). Multiple evanescent white dot syndrome following simultaneous hepatitis-A and yellow fever vaccination. Ocul Immunol Inflamm.

[4] Baharani A, Reddy RR (2023). Multiple evanescent white dot syndrome following adenovirus vector-based COVID-19 vaccine (Covishield). Ocul Immunol Inflamm.

[5] Inagawa S, Onda M, Miyase T, Murase S, Murase H, Mochizuki K, Sakaguchi H (2022). Multiple evanescent white dot syndrome following vaccination for COVID-19: a case report. Medicine (Baltimore).

[6] Soifer M, Nguyen NV, Leite R, Fernandes J, Kodati S (2022). Recurrent Multiple Evanescent White Dot Syndrome (MEWDS) following first dose and booster of the mRNA-1273 COVID-19 vaccine: case report and review of literature. Vaccines (Basel).

[7] Ng HW, Niederer RL (2023). Lightning can strike twice: recurrent multiple evanescent white dot syndrome (MEWDS) following both COVID-19 vaccination and subsequent COVID-19 infection. J Ophthalmic Inflamm Infect.

[8] Jain A, Shilpa IN, Biswas J (2022). Multiple evanescent white dot syndrome following SARS-CoV-2 infection - a case report. Indian J Ophthalmol.

[9] Munk MR, Stillenmunkes R, Tillmann A (2025). Evidence and consensus based imaging guidelines in multiple evanescent white dot syndrome multimodal imaging in uveitis (MUV) taskforce report 6. Am J Ophthalmol.

[10] Hang C, Yan Y (2022). Case report: visual snow as the presenting symptom in multiple evanescent white dot syndrome. Two case reports and literature review. Front Neurol.

[11] Lombardo J (2003). Multiple evanescent white dot syndrome and acute zonal occult outer retinopathies. Optom Vis Sci.

[12] Ng XL, Betzler BK, Testi I (2021). Ocular adverse events after COVID-19 vaccination. Ocul Immunol Inflamm.

[13] Bouhout S, Hébert M, Vadboncoeur J, Aubin MJ (2023). Multiple evanescent white dot syndrome following COVID-19 vaccines. Can J Ophthalmol.

[14] Gargouri MA, Yousfi N, Toutain J (2023). Multiple evanescent white dot syndrome following COVID-19 mRNA vaccination. Ocul Immunol Inflamm.

[15] Seong HJ, Lee CS (2022). Multiple evanescent white dot syndrome with submacular fluid in dome-shaped macula following COVID-19 vaccination: a case report. Korean J Ophthalmol.

[16] Gallo B, Talks JS, Pandit RJ, Browning AC (2023). Multiple evanescent white dot syndrome and choroidal neovascularization following SARS-COV-2 infection in a patient on dabrafenib and trametinib. Ocul Immunol Inflamm.

[17] Zeng Y, Du Z, Shao C, Zhao M (2024). Comprehensive insights into COVID-19 vaccine-associated multiple evanescent white dot syndrome (MEWDS): A systematic analysis of reported cases. Hum Vaccin Immunother.

[18] Goh D, Lim JC, Fernaíndez SB (2022). Case report: persistence of residual antigen and RNA of the SARS-CoV-2 virus in tissues of two patients with long COVID. Front Immunol.

[19] Menuchin-Lasowski Y, Schreiber A, Lecanda A (2022). SARS-CoV-2 infects and replicates in photoreceptor and retinal ganglion cells of human retinal organoids. Stem Cell Reports.

[20] Ng HW, Scott DA, Danesh-Meyer HV, Smith JR, McGhee CN, Niederer RL (2024). Ocular manifestations of COVID-19. Prog Retin Eye Res.

[21] Maamar M, Artime A, Pariente E (2022). Post-COVID-19 syndrome, low-grade inflammation and inflammatory markers: a cross-sectional study. Curr Med Res Opin.

